# Bayesian network-based framework for exposure-response study design and interpretation

**DOI:** 10.1186/s12940-019-0461-y

**Published:** 2019-03-22

**Authors:** Nur H. Orak, Mitchell J. Small, Marek J. Druzdzel

**Affiliations:** 1 Department of Civil and Environmental Engineering, Carnegie Mellon University, Pittsburgh, PA USA; 2 Department of Engineering and Public Policy, Carnegie Mellon University, Pittsburgh, PA USA; 3 School of Computing and Information Sciences, University of Pittsburgh, Pittsburgh, PA USA; 4 Faculty of Computer Science, Bialystok University of Technology, Białystok, Poland; 5 Department of Environmental Engineering, Duzce University, Duzce, Turkey

**Keywords:** Health risk assessment, Exposure-response, Bayesian networks, Measurement error, Toxicology, Environment, Environmental health

## Abstract

Conventional environmental-health risk-assessment methods are often limited in their ability to account for uncertainty in contaminant exposure, chemical toxicity and resulting human health risk. Exposure levels and toxicity are both subject to significant measurement errors, and many predicted risks are well below those distinguishable from background incident rates in target populations. To address these issues methods are needed to characterize uncertainties in observations and inferences, including the ability to interpret the influence of improved measurements and larger datasets. Here we develop a Bayesian network (BN) model to quantify the joint effects of measurement errors and different sample sizes on an illustrative exposure-response system. Categorical variables are included in the network to describe measurement accuracies, actual and measured exposures, actual and measured response, and the true strength of the exposure-response relationship. Network scenarios are developed by fixing combinations of the exposure-response strength of relationship (none, medium or strong) and the accuracy of exposure and response measurements (low, high, perfect). Multiple cases are simulated for each scenario, corresponding to a synthetic exposure response study sampled from the known scenario population. A learn-from-cases algorithm is then used to assimilate the synthetic observations into an uninformed prior network, yielding updated probabilities for the strength of relationship. Ten replicate studies are simulated for each scenario and sample size, and results are presented for individual trials and their mean prediction. The model as parameterized yields little-to-no convergence when low accuracy measurements are used, though progressively faster convergence when employing high accuracy or perfect measurements. The inferences from the model are particularly efficient when the true strength of relationship is none or strong with smaller sample sizes. The tool developed in this study can help in the screening and design of exposure-response studies to better anticipate where such outcomes can occur under different levels of measurement error. It may also serve to inform methods of analysis for other network models that consider multiple streams of evidence from multiple studies of cumulative exposure and effects.

## Background

**Fig. 1:**
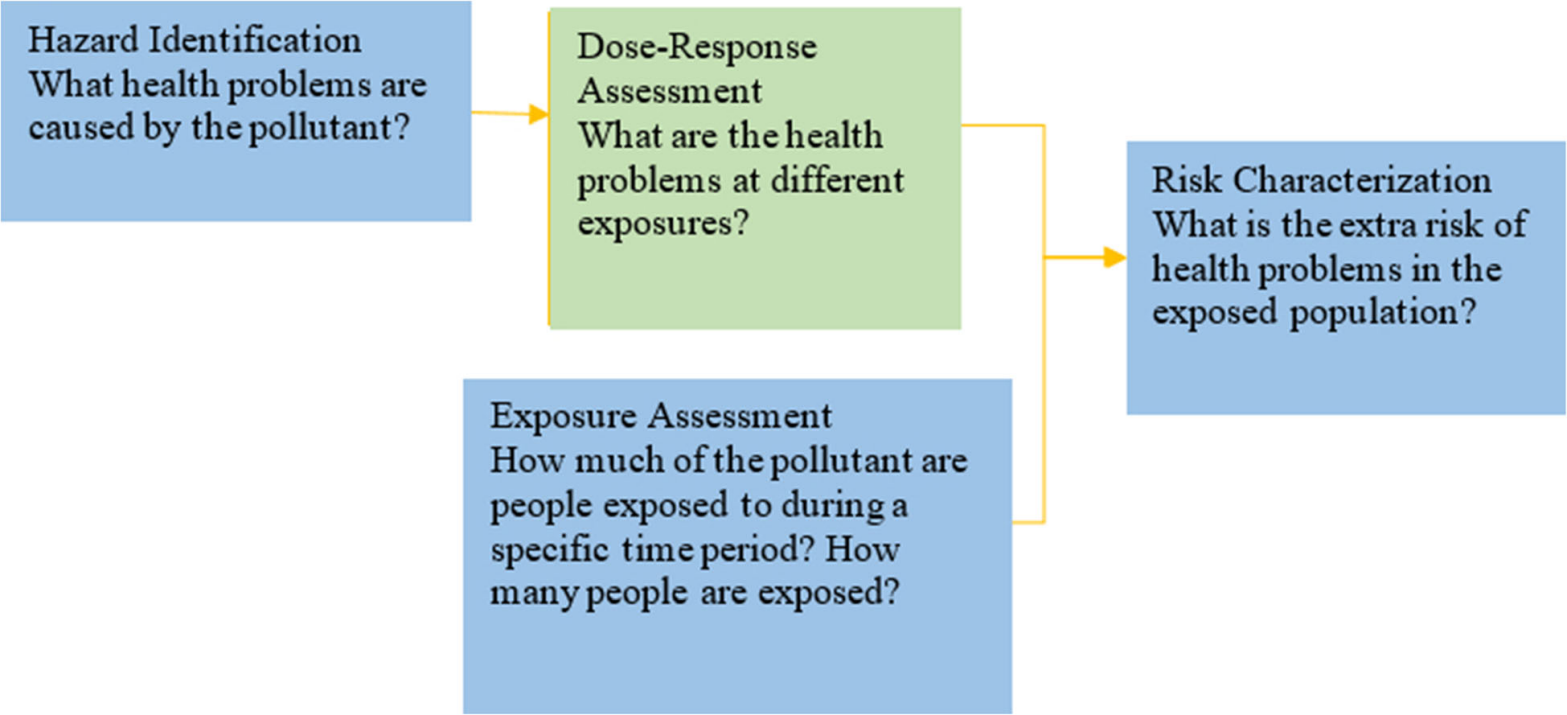
Components of the risk-assessment process (Source: https://www.epa.gov/risk/conducting-human-health-risk-assessment)

Exposure- and dose-response assessment are among the most critical steps of the environmental risk-assessment process (see Fig. 1). These provide information about the adverse health effects of different exposure levels in the population. In toxicological studies uncertainty is introduced due to experimental error (e.g., an imperfectly controlled environment, human factors and experimental conditions leading to dose variability, etc.); limited sample sizes; and the effects of high- to low-dose and animal-to-human extrapolation when interpreting the results of the study [1]. In epidemiological studies the assessment is uncertain due to exposure measurement errors; uncertainty in the relationship between exposure and dose to critical cells or organs; the influence of confounding factors affecting members of the population; and incomplete or erroneous data on health endpoints. In either case the relationship between the actual exposure level of a toxicant and the actual response is difficult to estimate by direct measurements [2–5]. The network model developed herein provides a direct, integrated method for assessing the value of such improvements in exposure and response measurement.

Toxicological experiments are generally done with high-dose compound exposure in laboratory animals, and these results are used to predict the potential adverse health endpoint(s) in humans, assuming that similar effects would be expected. However, the levels of chemical exposure in environmental settings are usually much lower than tested levels [1, 6]. Decisions about setting maximum contaminant limits can thus be biased by these measured responses at high dose. In epidemiological studies the sampled population and risk levels are often too small for the exposure-related increment to be statistically distinguished from background levels of the health endpoint. Epidemiological studies are also prone to known or unknown confounding factors which may affect estimation of exposure-response relationships in ways similar to the effects of measurement error [7–10]. Therefore, this study starts with key uncertainty problems in experimental studies: (1) How should prior knowledge be used to learn about the strength of the relationship between true exposure and true response? (2) How do measurement errors in exposure and response affect experimental design and interpretation for toxicological and epidemiological studies? and (3) What are the sample sizes needed to determine whether a significant exposure-response relationship is present?

We know that prior scientific knowledge about exposure and response mechanisms can lead to better design and interpretation of study results. Furthermore, better understanding of the sources of measurement error, options to reduce it, and its effect on subsequent inference can increase the likelihood of successful experimental designs for future trials and for clinical use. In order to achieve this goal, we propose a Bayesian network (BN) model-based approach to analyze the probabilistic relationship between true exposure and true response. BNs provide a simple yet holistic approach to the use of both quantitative and qualitative knowledge, with the distinct advantage of combining available information through a mix of expert judgment, mechanistic models, and statistical updating with observed outcomes [11–13].

Measurement error in statistical and risk science is a well-studied topic in the literature [14–18]. However, effects of measurement error on the strength of concentration-response relationships in toxicological studies have been limited. BNs can help to understand the effects of measurement errors on the magnitude of an exposure- or dose-response relationship. There are three effects of measurement error in covariates: (1) it causes bias in parameter estimation, (2) it leads to a loss of power for the prediction of a relationship, and (3) it makes structural analysis difficult [19]. Sonderegger et al. [20] investigated the effects of unmeasured temporal variation, and they suggest temporal variation in contaminant concentrations causes important bias in the exposure-response relationship.

In the next section, we discuss our model, giving background on BNs and our estimation of model parameters. In the following section, we apply the model using illustrative values of model input parameters. We then present our results and discuss further possible applications of our methods and results.

## Methods

Using BNs as a risk-assessment tool allows us to investigate and quantify the causal relationships between several interacting variables and outcomes because there is a theoretical relation between causality and probability [11, 21–23]. Therefore, we aim to predict the strength of relationship between True Exposure (*TE*) and True Response (*TR*) based on observations of exposure and response from studies with different sample sizes.

**Fig. 2:**
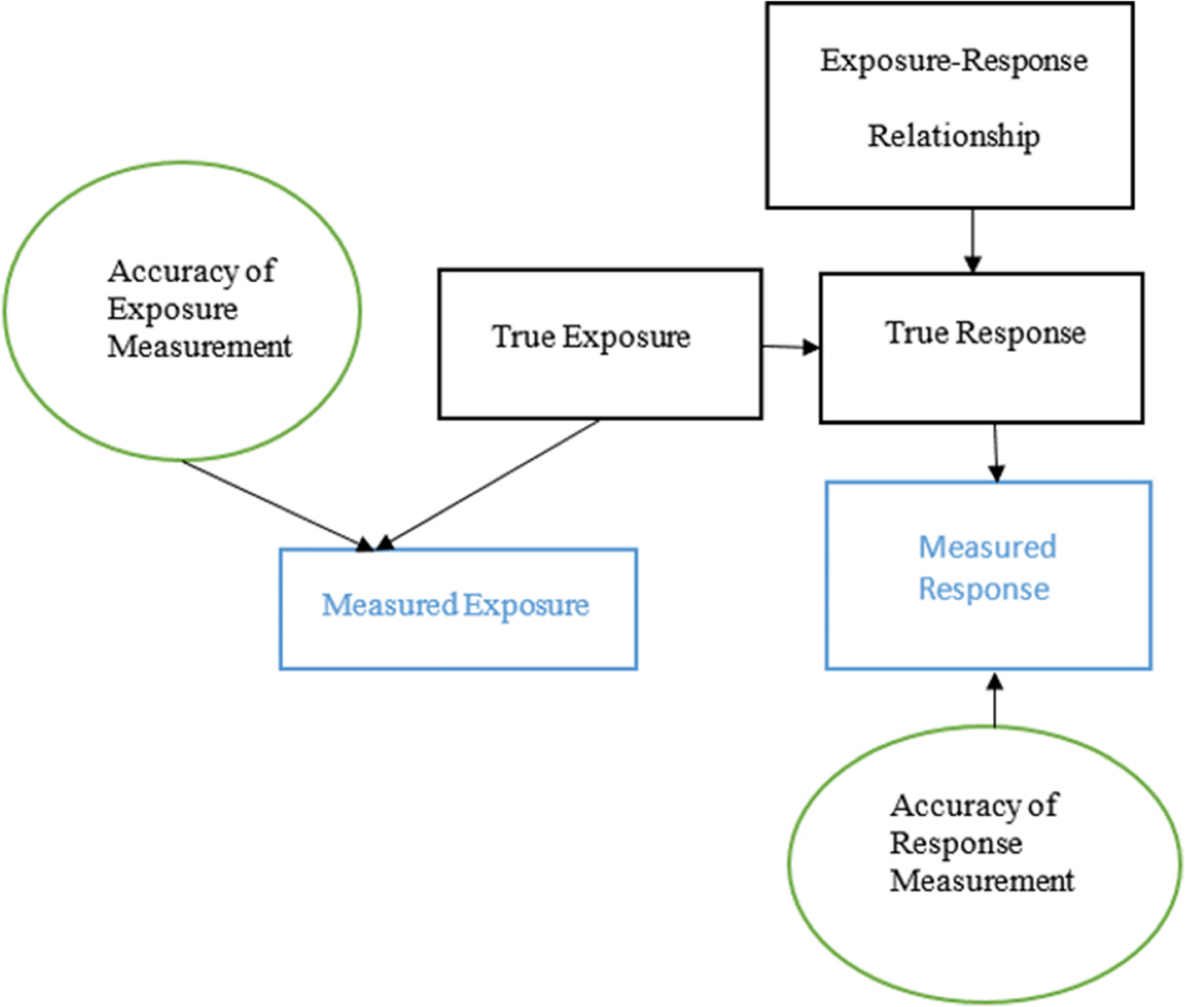
An influence diagram for a dose-response assessment

BNs capture cause-and-effect relationships through the structure of an acyclic directed graphs, so understanding and designing the diagrams is critical. Figure 2 shows the directed graph of a theoretical exposure-relationship assessment. This simplified influence diagram considers several sources of error under different nodes. Reductions in the *Accuracy of exposure measurement* (that is, greater errors in exposure measurements or classification) could result from incomplete spatial and/or temporal coverage of the target population in the exposure study; the selection of environmental or internal (biomarker) metrics of exposure that provide an imperfect indication of the critical exposures that matter to the health endpoint; and laboratory and field sampling errors for these metrics. Reductions in the *Accuracy of response measurement* (that is, greater errors in response measurements or classification) result from the occurrence of incomplete reporting or misdiagnosis of health endpoints in humans (for epidemiological studies) or laboratory animals (for toxicological studies); limited sample sizes in these studies; and errors in fitted relationships and extrapolations for response outcomes. *True exposure* and *true response* are the actual exposure and response levels in the target population, reflecting the true magnitude of the exposure-response relationship. These actual values are measured (or estimated) imperfectly to yield measured exposure and measured response.

### Bayesian networks

Bayesian networks were developed in the late 1980s to visualize probabilistic dependency models via Directed Acyclic Graphs (DAG) and model efficiently the joint probability distribution over sets of variables [11, 24]. BNs are strong modeling tools and are relatively simple compared to other modeling approaches [13]. The characterization of linkages between variables is typically probabilistic, rather than deterministic, so that BNs allow use of both quantitative and qualitative information [24].

BNs have been used to analyze problems, and to plan, monitor, and evaluate diverse cases of varying size and complexity in several different disciplines [25–29]. Bayesian models are particularly appropriate for environmental systems because uncertainty is inherent, and BNs have been used widely for ecological applications [30]. Similar potential exists in the field of human health risk assessment [31]. Specifically, a few studies have investigated the relationship between true exposure and true response through BNs [32–35]. Marella and Vicard (2013) [33] investigated the measurement error generating mechanism by developing an object oriented Bayesian network model. There are also a number of recent examples of BN and related DAG applications in health-risk assessment [21, 36–38]. Several studies investigated interactions among cancer risk components caused by environmental exposure by using a probability tree approach [39, 40]. These papers focus on exposure-response predictions as a part of fundamental assumptions of the cancer risk network.

Calculations in BNs are based on repetitive applications of Bayes’ theorem (also known as Bayes’ rule or Bayes’ law), which was first derived by Thomas Bayes and published posthumously in 1764 [41]*.* According to Bayes’ theorem, a prior probability provides information about the initial uncertainty of a parameter (before data are collected, based, for example, on expert judgment), while the posterior probability is calculated using the observed data and its likelihood function to update the uncertainty distribution of the parameter [42]. This feature of the theorem differentiates Bayesian statistical models from ordinary non-Bayesian statistical models because the Bayesian approach is a mixture of ordinary models and a joint distribution over the measured variables, and it may incorporate subjective prior beliefs [23]. Bayes’ rule (Eq. 1) allows for iteratively updating the marginal probability distribution over each node in the network as new data are collected and states in the network are observed [41, 43].

1 $$ p\left(X=x|Y=y\right)=\frac{p\left(X=x,Y=y\right)}{p\left(Y=y\right)}=\frac{p\left(X=x\right)p\left(Y=y|X=x\right)}{\sum_{x\prime }p\left(X={x}^{\prime}\right)p\left(Y=y|X={x}^{\prime}\right)} $$

BNs bring a holistic approach to understand the important pathways in networks, which are not easily expressed by mathematical equations, by integrating qualitative expert knowledge, equations, probabilistic modeling, and empirical data [11, 44, 45]. When the response variable (X in Eq. 1) is categorical, the BN provides the equivalent of a probabilistic classification approach [46].

**Fig. 3:**
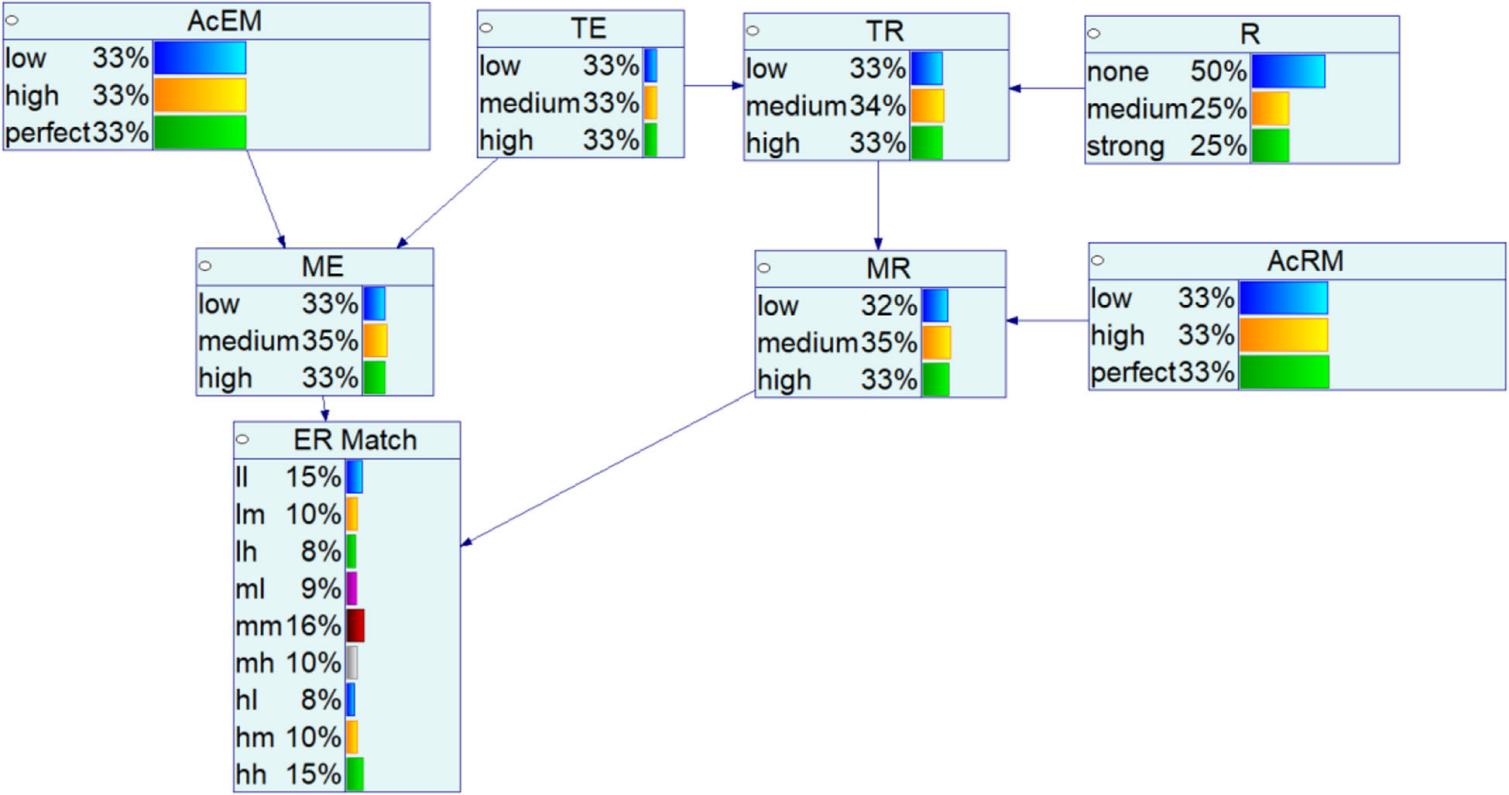
BN model for dose-response assessment with prior probabilities

We developed a BN (Fig. 3) based on the preliminary directed graph of Fig. 2 by using the GeNIe software package [47]. We chose this software because of its quality, flexible data-generation feature, its user-friendly graphical interface, and availability (free of charge to academic users). The default belief updating algorithm in GeNIe is the clustering algorithm, the fastest-known exact algorithm for Bayesian networks. The clustering algorithm was originally proposed by Lauritzen and Spiegelhalter (1988) and improved by several researchers [48, 49]. We chose the Estimated Posterior Importance Sampling (EPIS) algorithm for sampling cases, which provides more precise results compared to other available algorithms [47].

The accuracy of exposure-measurement and response-measurement levels are represented by *AcEM* and *AcRM*, respectively. These accuracy levels can be affected by errors at various stages of the exposure or response estimation activities, as described above. The measured (observed) values of exposure and response are termed *ME* and *MR,* respectively. The true exposure (*TE*) and true response (*TR*) values are the actual exposure and response levels. Node *R* represents the complex relationship between *TE* and *TR*. For instance, if *R* is strong, then the degree of causal influence of *TE* on *TR* is high and the association between *TE* and *TR* approaches a nearly perfect alignment. That is, low TE almost always yields low TR, medium TE almost always yields medium TR, and high TE almost always yields high TR. As such, an increasing strength of relationship (from none to medium to strong), indicates an increased health risk associated with increasing exposure. The state *none* represents the event that there is no causal linkage between true exposure and true response, so that increasing the exposure levels does not impart any additional risk of the targeted health effect.

The node *ER Match* is used to compile the results of an exposure-response study, with each subject in the study classified into one of the three exposure states (l, m or h) and one of three response states (l, m or h), yielding nine possible outcomes for *ER Match:* (*ME, MR*) = (l, l); (l, m); (l, h); (m, l); (m, m); (m, h); (h, l); (h, m); and (h, h). This outcome node can consider outcomes for individuals or groups of individuals, with resulting probability updates then propagated back through the network. When the measured exposure and measured risk are the same, i.e., states (l, l), (m, m), or (h, h), this lends support to the belief that a strong relationship exists between the true exposure and the true risk, especially when the measurement errors are low. When the states do not match, this lends support to the belief that the relationship is not strong, and possibly that there is no relationship at all (or the relationship is masked by measurement error).

In the application below we assume a sequence of scenarios for the exposure-response relationship and the measurement errors, and use these to simulate synthetic measured outcomes in a study population of a given size. These results demonstrate the statistical behavior of the network model and the probability that correct inferences will be drawn for each scenario, in particular showing the variability of inferences and the rates of convergence with sample size.

### Parameterization of the illustrative Bayesian network model

To provide an illustrative demonstration of the Bayesian network methodology, we select representative values of the conditional probability tables (CPTs) and prior probabilities in the network to demonstrate how measurement errors influence the ability to distinguish between the possible strengths of the exposure-response relationship: none, medium or strong. The critical CPTs in the model include those for:

i) the measured exposure, ME, as influenced by the true exposure (TE) and the accuracy of the exposure measurement (AcEM);
ii) the measured response, MR, as influenced by the true response (TR) and the accuracy of the response measurement (AcRM); and
iii) the true response, TR, as influenced by the true exposure (TE) and the strength of the exposure-response relationship (R).

The conditional probabilities in CPTs i) and ii) reflect the degree of correspondence between the true exposure and the measured exposure, and between the true response and the measured response, respectively. Tables 1 and 2 shows the CPTs for ME and TR, respectively. The first row of the table indicates the states of AcEM followed by the states of TE. For example, if AcEM = low, and the true exposure = TE = low, then the probability that the measured exposure, ME = high equals 0.2.

**Table 1:**
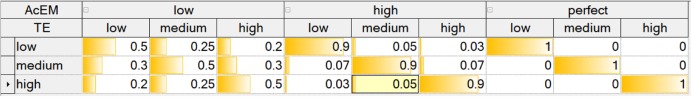
Conditional probability distributions for measured exposure, *ME (The first row represents the accuracy of exposure measurement, AcEM. The second row shows the True Exposure levels, TE. The first column categories (low, medium, and high) are for the ME node)*

**Table 2:**
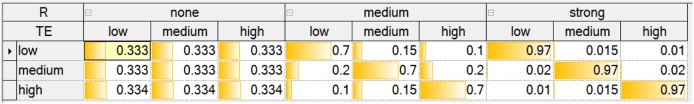
Conditional probability distributions for true response, *TR (The first row represents the strength of relationship, R. The second row shows the True Exposure levels, TE. The first column categories (none, low, medium, and high) are for the TR node)*

We assume that there is no prior information about the distributions of the top nodes in the network. Therefore, we use the uniform prior probability distribution over each variable, i.e., we assume that each state in a node with three outcomes has a 33% probability of occurrence, except the relationship (R) node. The *R* node prior probability is designed to investigate any potential relationship in addition to the strength of relationship. We thus assume a 50% probability of no existing relationship and a 50% probability of some relationship, allocated equally between a medium or a strong relationship, with 25% probability each (see Fig. 3). In all of the analyses that follow, “what if” scenarios are specified by choosing particular values of AcEM and AcRM, to determine the effect of different levels of measurement accuracy.

### Data simulation and analysis

We simulate random cases for nine scenarios (Table 3) using GeNIe which allows the users to generate random cases that are representative of the network based on the overall joint probability distribution of the nodes and their states. Each scenarios representing potential combinations of strength of relationship (R), the accuracy of exposure measurement (AcEM) and the accuracy of the response measurement (AcRM). To limit the number of scenarios considered, AcEM and AcRM were varied together so that scenarios reflect either low, medium or high accuracy for both the exposure and response measurements. We progressively increase the sample size from *N* = 1 to *N* = 1000 in the following examples, with the posterior probabilities following inclusion of case i serving as the prior probabilities for case i + 1.

**Table 3:** Nine scenarios for power evaluation

Simulation No	Scenario	
	Relationship (R)	AcEM - AcRM
1	None	Low-Low
2	None	High-High
3	None	Perfect-Perfect
4	Medium	Low-Low
5	Medium	High-High
6	Medium	Perfect-Perfect
7	Strong	Low-Low
8	Strong	High-High
9	Strong	Perfect-Perfect

GeNIe allows the user to generate random cases that are representative of the network, according to the joint probability distribution over the nodes and their states. Each case represents a hypothetical individual in a group of N that was exposed to a low, medium or high amount of toxicant in an environment, either with uncertainty based on the (equal prior) probabilities shown in the TE node in Fig. 3, or as specified for the scenarios below by selecting either low, medium or high exposure with 100% probability. A “true” population is thus simulated for a scenario with an assumed strength of relationship (none, medium, or strong) and specified levels of exposure and effect measurement error (low, medium or high for each). Given multiple sets of random cases with each (true) specification, we use each of the case sets to update a new “blank” copy of the network (that is, one with the prior specifications for the correct values of AcEM and AcRM, we assume to know the accuracies) and infer the posterior probability that the strength of relationship (informed by the case set) is none, medium, or strong. In essence, we use the simulated study results to update the assumed prior beliefs (in this case, uninformed) regarding the strength of the exposure-response relationship. If the inferred probabilities align with the true strength of relationship used to generate the cases, then we conclude that the simulated exposure-response study has the power to properly infer the strength of relationship. This power depends on the accuracy of the measurements and the sample size N, i.e., the number of random cases in each case set. As N increases, the power for proper inference likewise increases. In order to demonstrate the comparative results for different sample sizes, we simulated several N values: 20, 50, 100, and 1000.

**Fig. 4:**
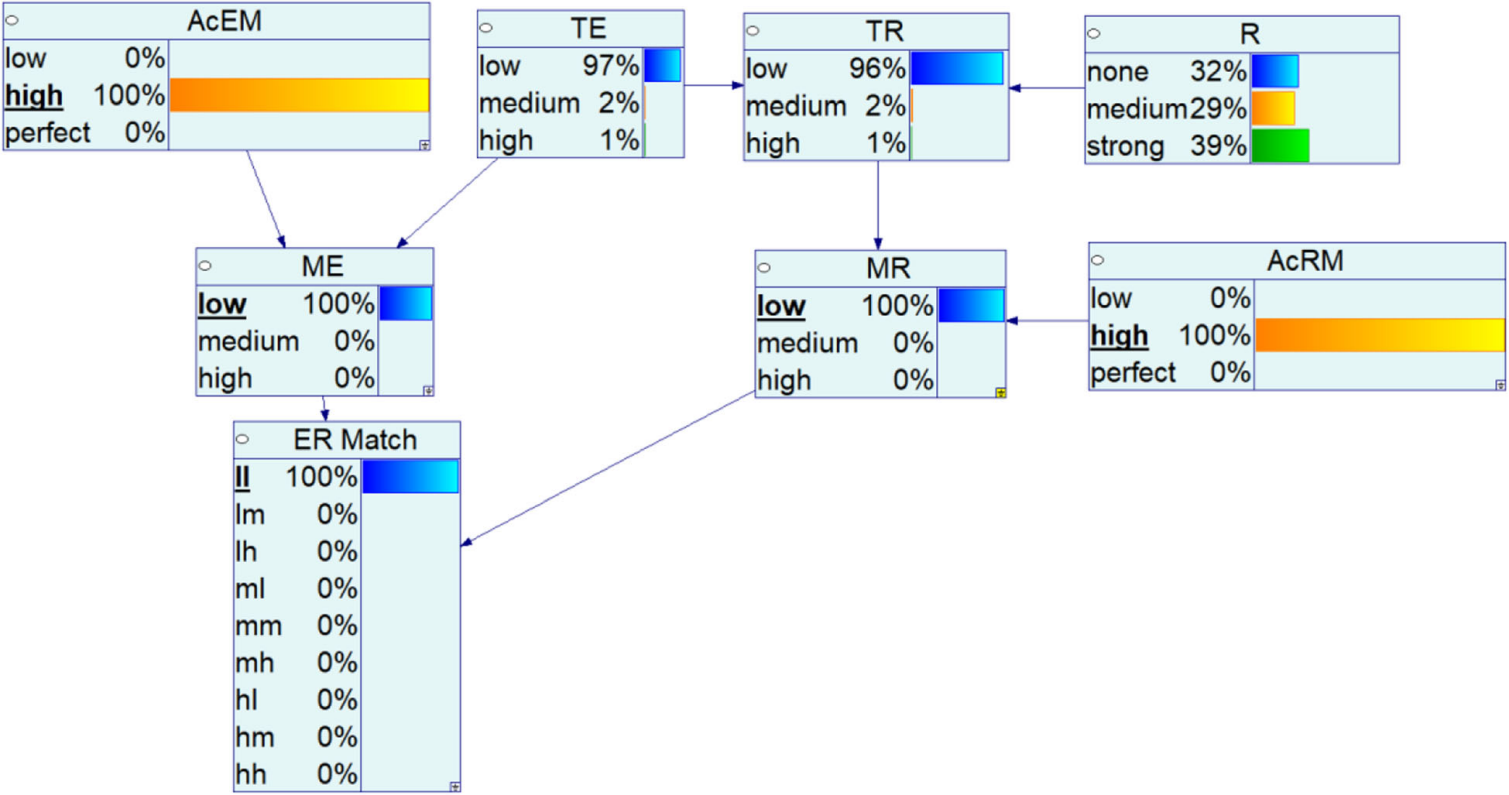
An example: updated BN model for *AcEM-AcRM*: low-low associated relationship (*R*) assessment and a single *ll* case

The following summarizes the steps in the simulation analysis:

1. Assign a true state for *R, AcEM*, and *AcRM* (e.g., define the scenario, Fig. 4, perfect-perfect, high-high, low-low),
2. Generate a synthetic dataset D of size N for the selected scenario, and repeat for 10 trials,
3. Count the frequency and calculate the average for each state of *ER Match*,
4. Calculate the posterior distribution for each state of *R*, given the specifications of the selected scenarios, and the sequential network updates calculated for each case in the dataset D, and
5. Repeat steps 1–4 for different sample sizes (N).

To implement sequential updates of the node state probabilities, we use the Bayes factor (BF) to facilitate the calculation. The BF is first computed as the likelihood ratio of a given set of states in the network relative to the other states, given the (simulated) data comprising *ER Match*. With a particular focus on the alternative states of *R*: Ri; i = 1,3, corresponding to a strength of exposure-response relationship of none, medium and strong, respectively, the Bayes factor is given by [50]:

2 $$ BF= Bayes\ Factor=\frac{likelihood\ of\ data\ in\  ER\  Match\ given\  Ri}{likelihood\ of\ data\ in\  ER\  Match\ given\ not- Ri} $$

An increasing BF indicates increasing evidence in support of state value i.

Once the BF is calculated for combinations of states and observations (i.e., for each of the three states of *R* and for each of the nine observation states of *ER Match*), each sequential observation of *ER Match* updates the state probabilities for *R* as:

3 $$ Posterior\ Odds\ (Ri)= BF\ast Prior\ Odds(Ri) $$

where *Odds (Ri) = P(Ri) /* [*1 – P(Ri)*]

One important advantage of the BF is that it is not affected by the prior probability at a given stage, nor by the sample size used to inform this probability. Once it is computed using Eq. 2, it may be used repeatedly in Eq. 3 to update the state probabilities in the network as new observations are collected (or simulated) and processed. In the following comparisons, we compute posterior probabilities for 10 realizations of each scenario using an independent sample of *ER Match* for each. This allows us to track the effects of measurement error on the estimated strength of relationship and compare them across equally plausible samples from a given population scenario.

## Results and discussion

**Fig. 5:**
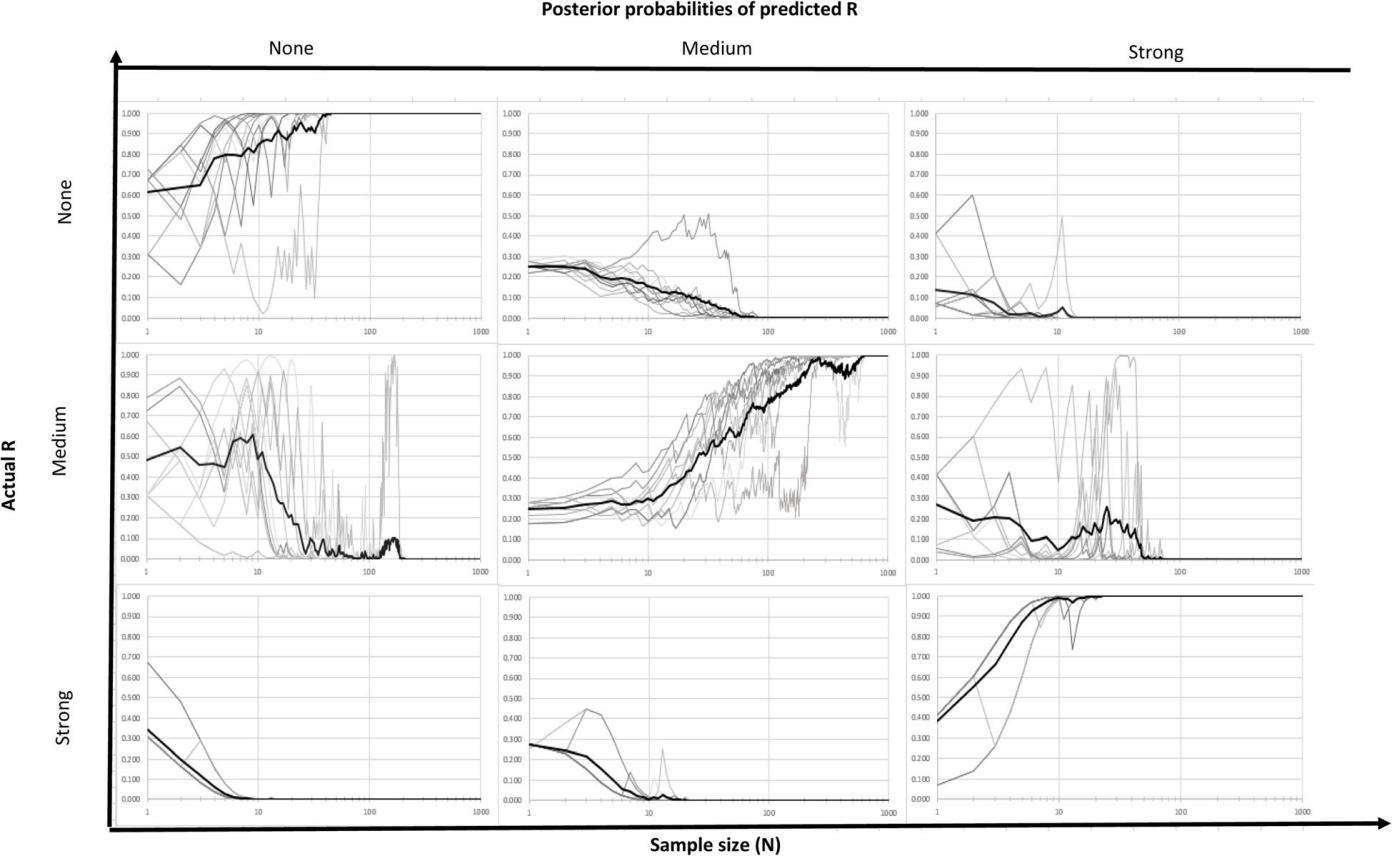
Posterior probabilities of different strength of relationship for the case of perfect-perfect accuracy level (title indicates the actual strength of relationship of dataset)

**Fig. 6:**
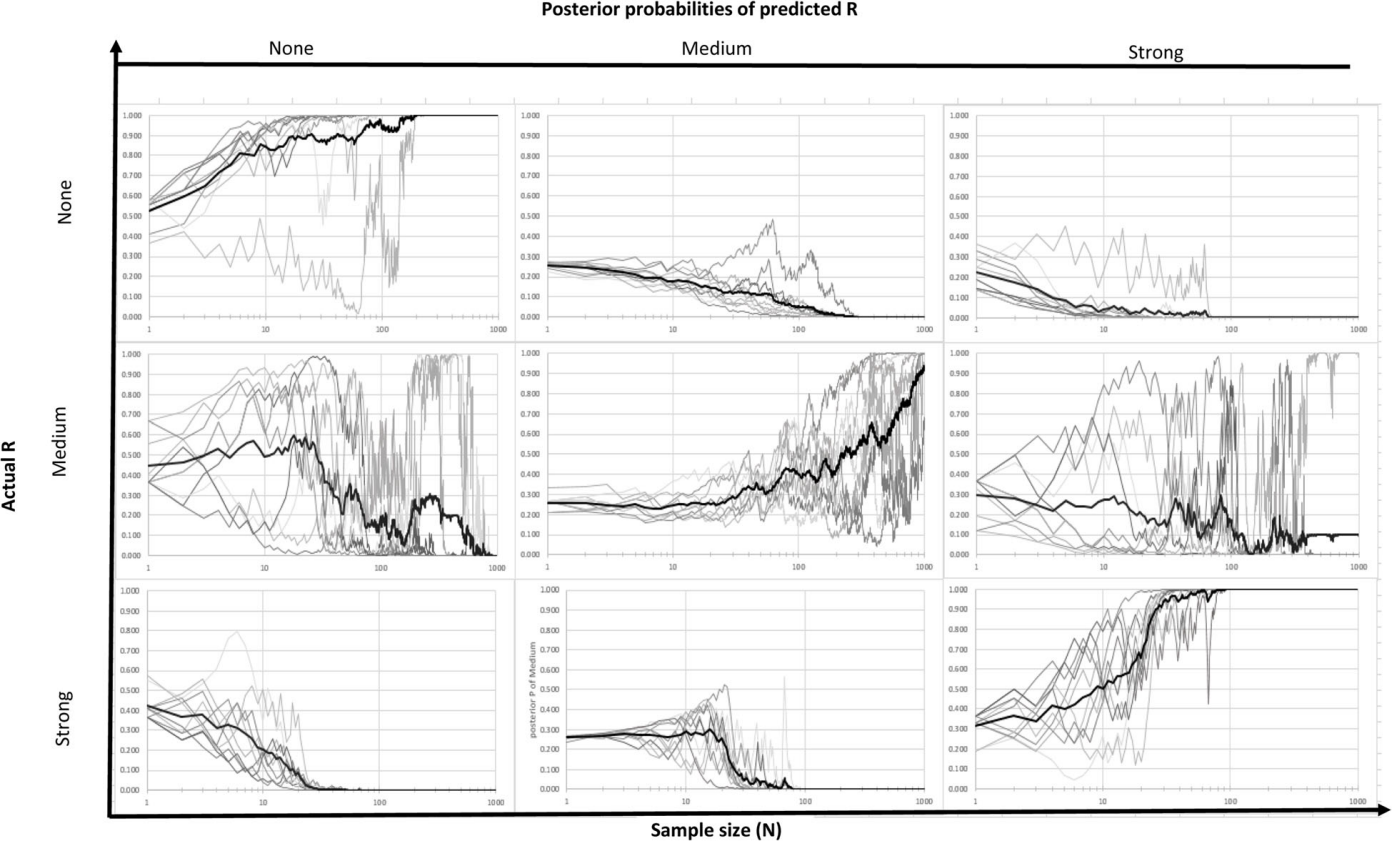
Posterior probabilities of different strength of relationship for the case of high-high accuracy level (title indicates the actual strength of relationship of dataset)

**Fig. 7:**
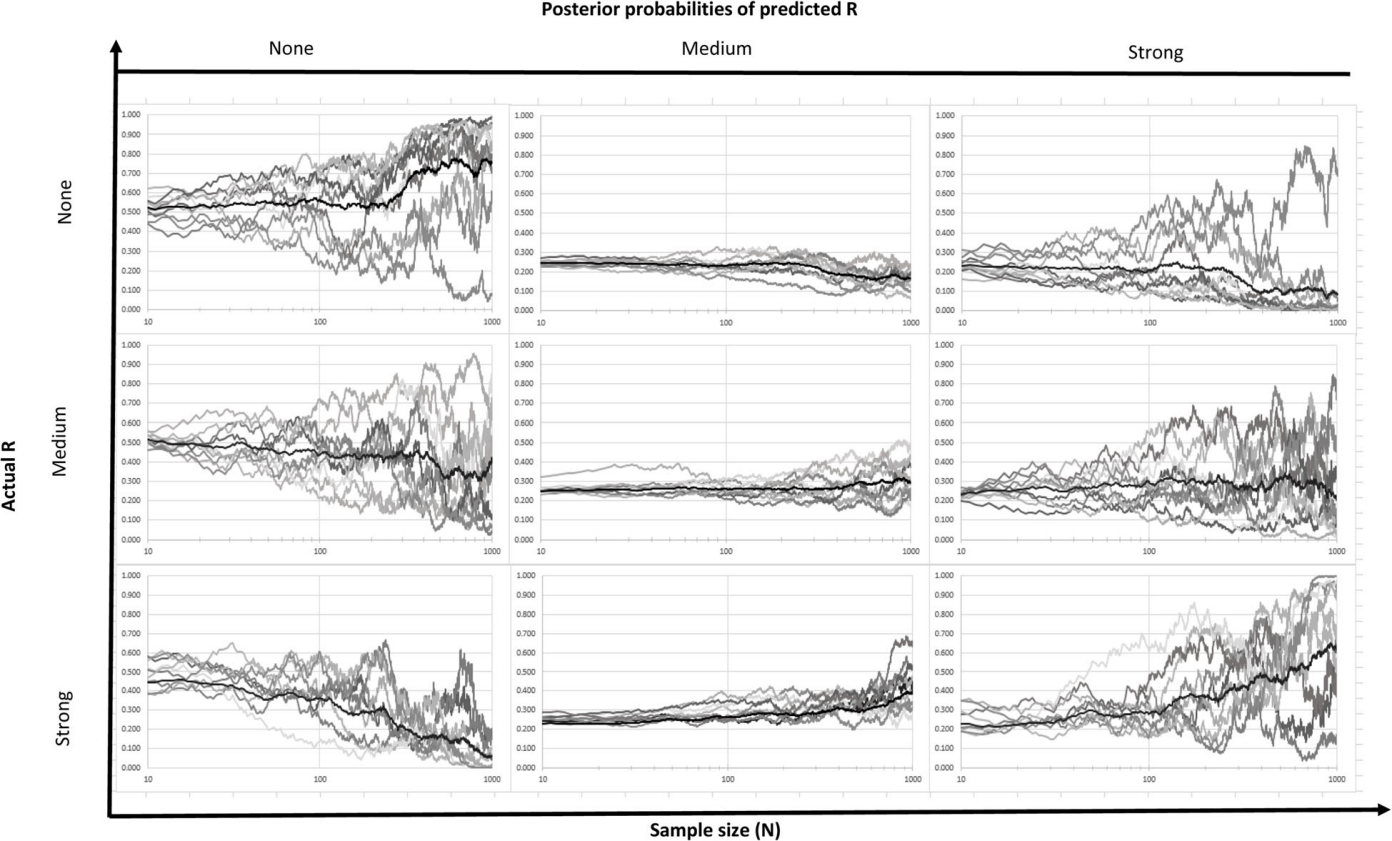
Posterior probabilities of different strength of relationship for the case of low-low accuracy level (title indicates the actual strength of relationship of dataset)

We evaluate the efficiency of the model by how well it predicts the strength of relationship when updated using synthetic *ER Match* results simulated for scenarios with specified values of R (none, medium, or high) and alternative scenarios for *AcEM* and *AcRM* (perfect-perfect, high-high, low-low). The results for these 3 × 3 = 9 scenarios are summarized in Figs. 5, 6 and 7, with the predicted probability for each of the categories of R shown as a function of sample size. In each case, one of the states for R is correct, corresponding to the original population designation, while the other two states are incorrect for the specified scenario. In each case the focus is upon whether and how quickly the predicted probability of the assumed true state of R approaches 1.0. Probability trajectories are shown as predicted from each of the 10 trials of simulated *ER Match* results for a given scenario (gray lines), as well as the mean probability prediction for each level of R across the 10 trials (black line).

In each figure, the rows represent the actual state of R used to generate the samples of *ER Match*, while the predicted posterior probabilities are for the state of R corresponding to each column. Each curve depicts the predicted probability of its column value of R given that its row state is true. The three plots along the diagonal of each figure show whether and how quickly the correct results are inferred by the network model using data with varying degrees of measurement error. The off-diagonal plots show whether, and for how large of a sample, false inferences are made for each of the two incorrect states.

Figure 5 summarizes the posterior probabilities of predicted *R* over different sample sizes assuming perfect measurements of both an individual’s exposure and their response. In this scenario, there is perfect correspondence between *TE* and *ME,* and between *TR* and *MR*, and the Bayesian network predictions for the true state of R converge to a probability of 1.0 in a relatively direct manner. This convergence is quite rapid for R = strong or none, occurring with approximate sample sizes of *N* = 20 or *N* = 50, respectively. Identification of R = medium is more difficult, requiring a sample *N* = 700 or more. Furthermore, as noted for many of the plots in Fig. 5, inferences from one or more of the individual trials (plotted in grey) exhibit divergent behavior well into the sample count, appearing as outliers relative to the other trials and diverging from the overall mean of the predicted probability over all or some of the pre-convergence sample sizes.

Figure 6 shows results for the high-high accuracy scenario where both the ME and MR correspond closely, but imperfectly, to TE and TR, respectively. As indicated, convergence for correct identification of the true R still occurs for all trials by an approximate sample size of *N* = 100 for R = strong, and by a sample size of *N* = 300 for R = none. For R = medium, convergence of all trials to a probability of 1.0 is still not achieved by a sample size of *N* = 1000. The overall slower convergence of the high accuracy vs. the perfect measurement scenarios is expected, as is the greater variance in individual trials exhibited in Fig. 6 compared to Fig. 5. The especially slow convergence for R = medium may result from our particular model parameterization, but also from the fact that the medium state for R is bounded on both sides by the alternatives none (below) and strong (above). If very strong evidence for R = none accumulates (with a very small number of samples where the subjects’ measured exposure and measured response align), this statistical overabundance of support for R = none still supports the subsequent inference that R = none. The same occurs for R = strong when there is a statistical overabundance (e.g., nearly all samples yield *MR* = *ME*). In contrast for R = medium, as unusual (perhaps non-representative) results accumulate, there is somewhere else for the fitted probability to go, either upwards to R = strong or downwards to R = none.

The effects of low-low accuracy (i.e., high measurement error) are illustrated in Fig. 7, where none of the true states of R and their associated samples lead to correct mean probability predictions that converge to 1.0 by *N* = 1000. For R = none and R = strong, the mean values of the probabilities are slowly progressing upward (reaching 0.7 for R = none and 0.55 for R = strong when *N* = 1000), but with extremely high trial-to-trial variation which grows larger with sample size. By the time *N* = 1000, a number of the trials for either R = none or R = strong predict the correct state with probability close to 1.0, but others predict the correct state with probability close to zero, providing “convincing” evidence for the wrong conclusion. Other trials predict probabilities for the correct state between 0 and 1.0, so that the inferences drawn from their exposure-response analyses span the range from correct to inconclusive to wrong. As such, from the results in Fig. 7, low accuracy measurements can cause significant mislearning to occur in many cases becoming more severe as the study size increases. The presence of variability for “None” and “Strong” cases allows for occasional high and low posterior probabilities compared to the “Medium” scenario.

To provide an overall summary of the effects of measurement error Table 4 shows the sample size needed to (on the average) infer with 90% posterior probability the correct strength (for the three true strengths of relationship) and the three accuracy levels. Increasing accuracy levels require smaller sample sizes to predict the strength of the true relationship. For instance, increasing the accuracy level from low to perfect causes a dramatic decrease in the required sample size (1000+ to 6) for the case of a *strong* relationship.

**Table 4:** The sample size needed to infer with 90% posterior probability of the correct strength

Accuracy Level	True strengths of relationship		
	None	Medium	Strong
Low	1000+	1000+	1000+
High	133	983	25
Perfect	32	205	6

The main goal of this study is exploring Bayesian network model as a tool to understand the effects of measurement and classification errors on the accuracy and precision of inferences drawn regarding the strength of exposure- and dose-response relationships. There is a high potential of applying the proposed method to different datasets. We acknowledge the limitations of this study. However, in the future, Bayesian methods can become a routine toolkit for assessing dose-response measurement and correcting measurement errors. Therefore, there is a growing need of scientific knowledge on advanced statistical methods. The proposed method provides important information on the prior knowledge and likelihood of a strong, medium or weak relationship; metrics of exposure and sources of exposure error or misclassification; and metrics of response and the possible causes of effects misclassification; and the additional data that would be needed to apply the method.

## Conclusions

New methods are needed to frame and quantify the joint effects of measurement errors and different sample sizes on the ability of exposure- and dose-response studies to properly infer the presence and magnitude of an actual epidemiological or toxicological relationship. DAGs can provide a powerful approach for visualizing dependencies between variables in a network, allowing the combination of expert judgment for measurement errors and the strength of a relationship with the quantitative study results.

We present an illustrative demonstration of a novel method to frame fundamental uncertainty questions in toxicological/epidemiological studies. We use BNs as a tool to understand the effects of measurement and classification errors on the accuracy and precision of inferences drawn regarding the strength of exposure- and dose-response relationships. For the parameters assumptions, differences in the power to properly infer a strong vs. medium vs. no relationship are found. The results show that cases where the actual strength of relationship is either R = none or R = strong are easier to predict (with smaller sample size) than the case where R = medium. In general, increasing the sample size increases the accuracy level for the predicted *R* for almost all scenarios, except when the measurement error is high (*AcEM*, *AcRM* = low). For these scenarios, the predictions, even over many trials, exhibit little or no convergence. Furthermore while improved measurement accuracy does increase the efficiency of *R* prediction on average (yielding faster convergence of the mean probability), in most scenarios there are a few, or in some cases many, of the 10 replicate trials that yield incorrect inferences even as the sample size becomes quite large. This suggests that environmental health scientists must be aware of the (perhaps surprisingly high) probability of incorrect inferences being drawn from a single exposure-response study. Extended versions of the network demonstrated here could assist in this assessment, including, for example, the effects of possible confounding exposures and behaviors, and inclusion of multiple sets of toxicological and epidemiological study results. These insights would be of value in a wide range of contexts requiring the design and interpretation of toxicological and epidemiological studies.

## Abbreviations

AcEM: The accuracy of the exposure measurement
AcRM: The accuracy of the response measurement
BF: Bayes Factor
BN: Bayesian Network
CPT: Conditional probability table
DAG: Directed acyclic graphs
ER: Exposure-Response Match
ME: Measured exposure
MR: Measured response
TE: True exposure
TR: True response
